# Suicide in Old Age: a threaten to Human Rights?

**DOI:** 10.1192/j.eurpsy.2022.124

**Published:** 2022-09-01

**Authors:** D. De Leo

**Affiliations:** Griffith University, Australian Institute For Suicide Research And Prevention, Brisbane, Australia

**Keywords:** Suicide, late Life, old age, prevention

## Abstract

Mental health care remains a pillar of suicide prevention also in late, but the range of interventions should include attention to the many socio-environmental conditions that are relevant to this stage of life. Community programs that promote a sense of worthiness and belonging should be strongly encouraged in order to preserve personal identity and social integration. Loneliness has to be counteracted in its many facets and with vigour, given its multiple negative impacts. The fight against stigma and ageistic views - still deeply rooted even among health workers – should be carried out with determination. Active promotion of a culture of resilience and adaptation to different phases of life and the changes imposed by them should constitute the founding bases of all efforts aimed at promoting a successful aging process. Combating discrimination against older people, as well as promoting basic social determinants of health, would help prevent suicide. However, these issues are still very underrepresented in the global agenda of health care. While having an effective impact on the numerous forms of discrimination would require legal interventions by governments, fighting stigma would primarily involve education aimed at changing beliefs and attitudes. Promotion of human rights, with particular regard to protection against abandonment, abuse and violence - particularly deleterious aspects in old age – appears as essential for personal empowerment of older individuals. Once become more powerful, older people may become more capable of defending their interests in terms of quality of life and protection against risk factors for suicide.

**Disclosure:**

No significant relationships.

